# Using a ‘rich picture’ to facilitate systems thinking in research coproduction

**DOI:** 10.1186/s12961-019-0514-2

**Published:** 2020-01-31

**Authors:** Kathleen P. Conte, Seanna Davidson

**Affiliations:** 1 The Australian Prevention Partnership Centre, Sydney, Australia; 2 Menzies Centre for Health Policy and University Centre for Rural Health, School of Public Health, Faculty of Medicine, University of Sydney, Sydney, Australia; 3 The Systems School, Melbourne, Australia

**Keywords:** Systems thinking, soft systems methodology, quality improvement, coproduction, translation, health promotion, health information technology

## Abstract

**Background:**

In coproduction research, traditional ‘end-users’ are involved in the entire research process. The aim is to facilitate research translation by improving the timeliness and relevance of research. Because end-users often come from multiple sectors and hold diverse perspectives and priorities, involving them in coproduction can be challenging. Tools and approaches are needed to support coproduction teams to successfully navigate divergent viewpoints while producing rigorous but meaningful research outcomes. Rich pictures are a systems thinking tool to help make sense of complexity. In this paper, we describe how we developed and applied a ‘rich picture’ in a coproduction project with policy-level partners.

**Methods:**

Guided by systems thinking principles, we conducted a systemic analysis of ethnographic fieldnotes collected as part of a broader study that examined the dynamics between an IT system and the implementation of the state-wide childhood obesity prevention programmes it was designed to monitor. Translating qualitative themes into metaphor and imagery, we created a visual depiction of the system to reflect the experience of the system’s users (health promotion practitioners) and facilitated a workshop with policy-level programme administrators (i.e. participants, *n* = 7). Our aim was to increase the transparency of the system for our research partners and to spark new insights to improve the quality of programme implementation.

**Results:**

Guided by provocative questions, participants discussed and challenged each other’s thinking on the current functioning of the system. They identified future lines of inquiry to explore for quality improvement. Participants strongly agreed that the picture was a constructive way to engage with the ethnographic data but were challenged by the information and its implications. The opportunity for participants to co-learn from each other as well as from the picture was an added value.

**Conclusion:**

In the context of the facilitated workshop, the rich picture enabled research partners to engage with complex research findings and gain new insights. Its value was harnessed via the guided participatory process. This demonstrates the importance that, in the future, such tools should be accompanied by practices that enable participants to think with and apply systems thinking concepts and principles.

## Background

Coproduction has been heralded as a solution to addressing complex problems. It brings together diverse groups and stakeholders to develop deeper understandings and innovate new solutions to complex problems [1]. Coproduction promises to improve the translation of research knowledge into practice by engaging end-users in the design, execution and interpretation of research [2], and it aims to ensure that research is meaningful, timely and relevant to the needs of end-users, thereby improving impact.

Formally defined as “*the joint working of people who are not in the same organisation to produce goods or services*” [3, 4], coproduction has been enacted in many forms and settings. This diversity means there is currently no consensus on what coproduction is, who it involves or how best to do it [3]. Evidence-based strategies for how to achieve desired outcomes via coproduction are lacking because evaluation research on coproduction processes is minimal [5, 6]. Importantly, strategies on how to involve partners in later research stages – particularly the data analysis stage – are lacking [7]. Involving stakeholders in data analysis and interpretation presents time restraints and requires technical expertise to conduct analyses. Involving stakeholders in qualitative analysis raises ethical challenges in that sharing quotations or field notes – even if de-identified – may cause participants to be recognised. From project set-up phase to final dissemination, coproduction teams must navigate potential tensions, created and exacerbated by the divergent priorities and expectations of the multiple stakeholders involved [8–10].

Yet, it is this congregation of diverse perspectives that gives coproduction profound potential. Co-analysing and co-interpreting data with stakeholders may yield many benefits including a deeper, shared understanding of the system and the ability to identify immediate actions to advance change. Additionally, because coproduction partners are often embedded in context, they can offer insights that may otherwise be unapparent or overlooked by researchers. Therefore, serious attention and investment is needed to develop and evaluate tools and processes that could assist teams to better work in coproduction.

To this end, systems thinking approaches hold great promise because they embrace the complex messiness that arises between different perspectives and stakeholders and provide tools and methods to do so [11, 12]. Systems thinking is a way of making sense of complexity. It is comprised of (1) theories that provide concepts for understanding complexity, including attention to relationships, boundaries and perspectives; (2) tools and approaches that provide methods for exploring complex systems and problems; and (3) practices, undertaken by individuals or groups, through which capacity to think and act systemically is built by reflexivity and continuous learning [12, 13].

One of the key tenets of systems theories is that there are multiple actors that comprise a system, and each actor has a unique perspective about how the system works [13]. Because systems are dynamic, emergent and interdependent, engaging the insights of multiple actors can enable a better, more comprehensive understanding of the system itself [11]. In this way, systems thinking aligns with coproduction’s focus on bringing together multiple diverse actors, making coproduction a potential vehicle for systems thinking in-action. Like coproduction, systems thinking has been heralded as a solution to addressing complexity [14, 15]; however, like coproduction, it too suffers from a lack of evidence about how to meaningfully apply systems thinking principles in practice [16, 17]. To date, literature on systems thinking remains largely theoretical, with comparatively few examples of how to do it in practice to achieve the desired outcomes [17, 18]. That the fields of systems thinking and coproduction both lack evidence regarding how best to instrumentally do them likely illustrates that, as a field, we are not yet matured in our language, methods and processes to effectively capture and document complexity in action.

In this paper, we begin to address these gaps. Our purpose is to describe how we developed and used a systems thinking tool, called a ‘rich picture’, in the context of a coproduced ethnographic study examining a large-scale health promotion information technology (IT) system. By developing a rich picture, our aim was to increase the transparency of the ‘system’ we were studying by involving our partners in the interpretation of and reflection on research findings.

### Rich pictures

People have been using pictures and drawings to communicate complex concepts and ideas throughout the ages [19]. More recently, researchers are using art and pictures as part of research processes to communicate findings to diverse audiences [20, 21]. In the academic field of systems thinking, the concept of a ‘rich picture’ was developed by Peter Checkland [22] as part of his Soft Systems Methodology (SSM). SSM is an approach to exploring complex problems and facilitating change among people who hold different views, goals and agendas. In SSM, rich pictures are used as tools to make sense of a system and its behaviour. Practically, rich pictures are unstructured and non-linear representations of a situation that depict complexity. The actual picture itself can be simple, like a back-of-the-napkin drawing, or complicated, for example, by using graphic facilitation artists or mapping tools.

A rich picture offers a ‘map’ of a system. Where and how visuals are placed in the picture helps to orient the viewer to what is happening. It allows the whole system to be seen at once, even if only peripherally, which can enable patterns across the system to be surfaced and identified. In viewing, parts of the picture can be examined in detail, while also allowing parts to be seen in relation to one another. Thus, a rich picture can provide an ‘entry point’ to a complex system that has many facets.

Importantly, the rich picture is not meant to be an objective representation of a research finding. Therefore, its use requires two cycles of sense-making. In the first instance, sense-making occurs during the qualitative data translation stage when written data is being translated to imagery and metaphor. Second cycle sense-making occurs when participants interact with the visual through the lens of their own experiences and perspectives. Therefore, the value of a rich picture is not in its ability to concretely represent an external reality, but in its use as a tool to prompt second-cycle sense-making, to surface deeply held views, and to generate reflection and conversation that reveals new insights.

In systems thinking literature and SSM, rich pictures are generally created by the participants or groups themselves early in an inquiry process as an initial orientation and sense-making activity [22]; it is not an end in-and-of itself. Through the creation process, various understandings of the system become exposed and reveal the mental models of the pictures’ creators, including the emotional or political elements of a system [13]. However, rich pictures may also be used throughout an inquiry process [23, 24]. Crowe et al. [23] initially generated a rich picture from interview data. They added to and refined it over the course of a research project as new details about the system emerged. The rich picture became one of several data sources used to engage different stakeholder groups to share learnings and develop recommendations to enhance health service delivery.

Our use of a rich picture was in line with this latter approach. Our rich picture was developed from ethnographic data. We drew on traditional qualitative analysis techniques to develop the imagery by coding data and developing organising schemes in an iterative process of analysis in order to identify the key components of the system. We used symbols, imagery and metaphor to illustrate the system and how it operates [24]. However, similar to SSM, our picture was not intended as a final research product but rather as a tool through which to engage stakeholders in reflection, interpretation and further inquiry.

### Context

#### Overarching research project

The data for our rich picture came from a multi-site, ethnographic study of an electronic monitoring system called the Population Health Information Management System (PHIMS). Similar to electronic patient records in clinical settings, PHIMS was developed to track and support the implementation of childhood obesity prevention programmes and policies delivered by the health promotion workforce of New South Wales. Reported elsewhere, the main purpose of the overarching study was to explore the dynamics between PHIMS and health promotion practice (for more details see, e.g. [25, 26]).

We adopted a broad view of the field in which PHIMS was only one of multiple parts that make up a complex system of health promotion work and delivery within the overarching New South Wales Health system. There are many actors and departments within New South Wales Health, reflecting various disciplines and priorities. For this study, we worked with locally based teams of health promotion practitioners, state-level coordinators, IT designers, and policy-makers. Each of these actors represents different departments within the larger health system, plays specific roles and has different relationships to one another, as outlined in Table 1. We therefore consider the ‘PHIMS system’ as the broader set of interrelated roles, actors (of which PHIMS may be thought of as one, i.e. see [27]) and drivers that come together with the purpose of delivering childhood obesity prevention programmes across the state. PHIMS is both the central focus of this system and the boundary for this research.

**Table 1:** Roles of actors involved in the Population Health Information Management System (PHIMS) system and research project

Roles	Responsibility	Relationship to PHIMS	Relationship to the research	Relationship to other roles in the system
Health promotion practitioners	Deliver obesity prevention programmes in local health districts across the state	Uses PHIMS regularly to report progress and document other aspects of programme delivery	Other than one representative who was involved in the coproduction team, practitioners were research participants in the ethnographic study	Involvement with other roles in the system is hierarchical, with managers or directors having greater access to other teams, state-level decision-makers, etc.
State-level programme coordinators	Oversee, coordinate and support the work of the 14 health promotion teams that deliver obesity prevention programmes in local health districts	Can access aggregated PHIMS data about each teams’ progress towards achieving key performance indicators	High-level representatives were part of the coproduction team; operational team members participated in workshop	Acts as a go-between between all the other roles described here Facilitates communication and information sharing from higher-level decision-makers to the health promotion teams
Information technology engineers and analysts	Build, maintain and support the PHIMS IT system and its operations	Provides technical assistance to a range of PHIMS users	High-level representatives were part of the coproduction team; operational team members participated in workshop	Supports state-level programme coordinators and practitioners to use PHIMS
Policy-level decision-makers at the Ministry of Health	High-level decision-makers responsible for setting state-level policy and programming	Receives high-level progress reports on programme implementation derived from PHIMS data	Coproduced the research project, did not participate in the workshop	Coordinates with high-level decision-makers in each of the other departments

The overarching study was coproduced with a team comprised of representatives reflecting different roles in the system (as described in Table 1). The research partners co-developed the main research questions to explore how PHIMS was used on the ground, and to better understand the impact of PHIMS on practice. The parent study adopted an approach akin to focussed multi-sited ethnography, conducted over 2 years [25]. Multiple data sources were collected for the overarching study (i.e. interview recordings, pictures, etc.), but the ethnographic fieldnotes were the main data source we used for the rich picture. Fieldnotes were collected through 1–5 days of immersion with each of the 14 health promotion teams in the state of New South Wales, resulting in a data set of over 500 pages of single-spaced, typed notes.

#### Identifying the need to translate ethnographic data for coproduction and this sub-study

Because our partners were high-level policy decision-makers, we often met with their operational teams to discuss emerging findings and gain insights to inform the research. As the project progressed, these operational teams raised an interest in gaining more insights from the project that could be used to guide their operational work. The ethnographic team considered how best to share insights with partners and their teams throughout the entire project, particularly because of the need to maintain the anonymity of practitioners and sites. We also wanted partners to gain a better understanding of the health promotion practitioners’ experience of PHIMS and their role in the broader PHIMS system, and so saw this as an opportunity to increase the transparency of how the PHIMS system functions. We used several approaches, including regular project meetings, where we presented and discussed initial findings in more traditional forms (e.g. through PowerPoint presentations and academic paper drafts), developing ethnographic vignettes, and developing mock abstracts [28]. While these approaches were useful, the lead author of this study (KC) found that we needed a complementary process by which to invite the partners and their teams to see themselves as part of the system we were studying to better engage with the findings and implications. We chose the rich picture approach with the intent that it was an engaging, inviting way to prompt this reflexivity. Therefore, it was one of the several approaches (described above) we used to familiarise our partners with the data, interrogate it and thus support them to contribute to coproducing interpretations for final publications. The picture development and workshop occurred after ethnographic fieldwork was completed, but prior to the publication of research findings. In this paper, we describe the process of developing the picture, trialling it and conducting a 1-day workshop within this time period. Participants (*n* = 7) for the study reported here are comprised of an approximately equal proportion of staff members from two operational teams that oversee PHIMS and the obesity prevention programmes it monitors (Table 1). We drew on the Standards for Reporting Qualitative Research for the study presented here [29].

## Methods

### Consultation to identify lines of inquiry

We held several meetings between the researchers and the participants to explore what potential questions could be asked and the restrictions on what could be shared, and discussed potential options for moving forward. To facilitate this process, we shared the project’s codebook (including names of codes, definitions and number of quotations) so that participants could use the codes to identify topics of interest for deeper exploration. In Table 2, we report the co-developed purpose and aims guiding this inquiry, which was then used to frame and bound the rich picture.

### Content analysis

To develop the rich picture, a researcher from the overarching study (KC) and a researcher with expertise in systems thinking (SD) used an approach similar to a directed content analysis [30] using the co-developed purpose (described above) to focus the analysis. Using the codes selected by the participants, we used NVIVO to extract data and analysed it for patterns, relationships and dynamics between parts in the system. We kept detailed notes to develop an organising structure and summary of 13 categories representing the key findings (outlined in Table 3). Several iterations of coding were performed, and a process of reviewing, checking and discussion was used to clarify summaries and verify findings. Our aim was to visually depict a wider perspective of the system, i.e. a ‘scaled out’ view. Therefore, in each iteration, we synthesised findings and moved away from description towards abstraction [31]. To make decisions about the most significant and meaningful content to portray in the graphic, we constantly referred back to the co-developed aim, that of increasing the transparency of the system.

**Table 2:** Co-developed purpose and aims of inquiry and workshop process

Purpose of the overarching inquiry (i.e. what do we want to know?):	
To identify, describe, visualise and explore the non-technologically oriented processes that are built into and accompany the use of PHIMS. Topics to focus on include the ease and flow of information through the PHIMS system, where and how information is used and by whom, and the function and purpose of different aspects of the PHIMS design. As part of the workshop, participants will surface their assumptions and perceptions (e.g. mental models) regarding how PHIMS exists alongside these processes and, in turn, how these processes reinforce PHIMS use.	
Aim of the process (i.e. why are we doing this?): To increase the transparency of the PHIMS system.	
Specific goals (i.e. what do we want to accomplish?):	
1. Generate shared insight and understanding of PHIMS system as it currently is understood and used by Health Promotion Officers in practice.	
2. Present a visual depiction of the PHIMS system as understood via our research, i.e. a ‘systems map’ to be further refined in the workshop setting.	
3. Create a space to explore opportunities for quality improvements in practice PHIMS design/use and accompanying non-technological processes.	

### Developing the rich picture

The authors conducted a full-day workshop to review the final analysis and summaries and to design the picture. We tracked our process of design throughout the day to capture our brainstorming, ideas that did/did not work, considerations to inform the follow-up workshop, and potential issues that required revisiting and refinement. The steps are reported in Table 4. The main focus was on developing metaphors and symbols to best represent the main themes. We also considered alternate interpretations that the metaphors and/or imagery might provoke to identify potential unintended interpretations. We finished the day with a rough drawing of the picture, and a detailed description of the important visual components with their intended meanings. This summary document was sent to a graphic designer who created the final picture of the system. We worked with the graphic designer to refine the imagery in detail, including the placement of components in relationship to each other, the specific wording used, and the colours used.

**Table 3:** The PHIMS System of Practice from analysis of ethnographic data

1. Components of PHIMS System of Practice	
1.1 System rules – Rules that outline how the PHIMS system is supposed to work	
1.2 Data – Refers to the process of data entry in PHIMS, including the user-interface	
1.3 Contact Management – Regards the keeping and use of records for site contact details	
1.4 Reports – Reporting function in PHIMS and how PHIMS data is reported	
1.5 Scheduled follow-ups – How PHIMS prompts practitioners to engage with sites and how practitioners do so	
1.6 Health promotion practice – How principles of health promotion practice are reflected or enacted in the system	
2. Dynamics between parts of the system	
2.1 How used – How practitioners effectively use PHIMS (may be beyond intended use)	
2.2 Incongruence – Where there is dissonance between the demands from PHIMS and practitioner’s use of/or engagement with PHIMS	
2.3 Workarounds – How do practitioners get around system constraints or problems	
2.4 Missed opportunity – Instances where practice could be supported by PHIMS, but currently is not	
2.5 Black Box – Data goes into PHIMS, but then what happens?	
2.6 Blocks – Issues and challenges to intended functions of PHIMS or health promotion	
2.7 Fears – Expressed fear or concern regarding use and purpose of PHIMS	

### Trialling the rich picture

Because viewing a picture is a subjective experience, we trialled the rich picture to seek alternate perspectives and triangulate interpretations. Drafts of the picture were shared in-person with several members of the broader research team. Through an unstructured conversation, we explored what components were provocative, confusing details and the key messages that emerged. This provided an opportunity for researchers familiar with the broader data set to reflect on the metaphors and consider the extent to which they authentically represented the data. We found the researchers’ questions and comments of the visual to be useful in considering how others might respond and/or what aspects might be confusing. Given that each person has different perspectives of the system due to their role and experiences, each raised different interpretations and meanings. Therefore, this was a highly interpretive exercise rather than a process of ‘validating’ the picture. We had a similar experience when we presented the picture to one of the health promotion teams for feedback and review. They too compared and contrasted their interpretations of the visual with one another in a rich, reflexive discussion, but did not identify high points of conflict or contention over the depictions. Ten individuals provided feedback.

### Creating the interactive workshop

To facilitate second-cycle sense making, we designed an interactive workshop guided by the overarching goals (as described in Table 2). Both authors are experienced group facilitators and drew on these skills to design the process and to manage group dynamics during the workshop itself. We identified what we termed ‘design considerations’ (Appendix 1 in Table 5) or contextual factors that required attention when shaping the final workshop. For example, an important consideration was that we wanted to create an atmosphere of exploration and creativity. A second design consideration was who would participate in the workshop. While not ideal, it was not logistically feasible to involve the health promotion practitioners. Therefore, we designed the introduction and activities to acknowledge and consider this missing perspective and identify future issues to explore with them. By using the analytical questions we identified during the process of creating the visual and the key design considerations, reminding ourselves of the purpose, aim and goals of the work, and identifying where we wanted participants to land at the end of the day, we designed a series of activities to facilitate collective reflection and inquiry among the participants (a full workshop outline, including rationales for each activity are provided in Appendix 2 in Table 6).

**Table 4:** Summary of steps to develop the rich picture

Orientation and grounding	
1) Orient to the task by revisiting the aims and objectives of the work overall	
2) Identify who the process is being designed for, who will participate in the final workshop and what their relationships is to the topic	
3) Identify initial ‘design considerations’, i.e. aspects of the context and participants that must be considered when designing the final product and/or workshop; for example, how much time is available for the workshop? How big can the visual be? Can it be printed in colour?	
Documenting the process, including boundaries and limitations	
4) Track potential cautions to keep in mind in the design; for example, an important design consideration was that we wanted the activity to be one of discernment and inquiry vs. one of judgement and critique	
5) Throughout the day, question and re-question what a meaningful outcome of the workshop would look like	
6) Clarify boundaries to determine content that should be included and what is beyond scope; document these boundaries	
7) Capture analytical questions throughout the process that can be used to drive the workshop itself	
Analysing data to develop metaphors and imagery	
8) Review initial analysis in which data have been sorted into broader categories	
9) Use sense-making to identify key patterns in the data, i.e. ask ‘what is this a category a grouping of (more broadly)?’ and ‘how do these groupings sit alongside and interact with one another?’	
10) Use key patterns to develop a metaphor for visualisation. Experiment with multiple metaphors, consider the limitations of each and how the metaphor can be refined to best reflect the insights	
11) Repeatedly check back with the data and with the ethnographers’ broader knowledge of the field to ensure accuracy, thoroughness and authenticity	
12) Constantly test the developing metaphor to optimise accuracy and meaning, refine visualisations and ensure the focus/purpose of activity is maintained	

The first part of the workshop focussed on establishing a space for participants to engage with the picture from a place of curiosity and exploration. Initial activities aimed to encourage participants to take ownership of this process by reminding them that they had initiated this inquiry and chosen an interactive workshop as a means of engaging with new materials. The activities prompted reflection on why participants were interested in the inquiry topic, and why they requested taking a new approach. Subsequent activities encouraged participants to reflect on what they already do and do not know about the system to further the sense of exploration and openness to new information. The aim of these activities was for participants to consider their own knowledge of PHIMS before considering the perspectives of the health promotion officers that are presented on the map. The grounding and introductory work was also important in providing participants an opportunity to acknowledge that there are gaps in their knowledge – as it is impossible for one person to know everything about a complex system.

We then introduced the rich picture and encouraged participants to consider whether information from it filled gaps that participants had identified in their knowledge. Through this process, we aimed to create a boundary on what the picture could answer (as it is impossible to answer all questions), and what new information it provides. Importantly, this process was done in silence to allow individuals a chance to interact with information, process it and generate impressions free from the influence or opinions of others.

We developed a series of provocative questions to guide participants in reflection and group discussion to interrogate and unpack the picture in more detail. These questions purposefully directed participants to consider certain components depicted in the picture and their relationship to other components and to the picture as a whole. By moving between individual details, the full picture and participants existing knowledge, the process did not aim to generate consensus but rather to develop new insights and ask new inquiry questions.

### Analysing workshop data for further insights

During the workshop, participants engaged in several interactive activities that produced drawings, notes about insights, potential areas of future inquiry and other written outputs. This data were transcribed verbatim into electronic files (e.g. word documents and using Scapple to reproduce how sticky notes were laid out in relation to each other) and recordings of group conversations were transcribed verbatim. The transcripts captured conversation points that groups did not write down. An ethnographer from the overarching study observed and took fieldnotes, capturing her feel of the workshop and the engagement of participants. One-week post-workshop, participants were sent an online survey consisting of Likert-type questions about the extent to which objectives were met (range 1–6 where 6 indicates strong agreement) and open-ended questions. All participants responded. For this paper, we analysed the data described above to examine the extent to which the workshop met its objectives. In addition, we engaged in reflective dialogue and reviewed all data in-depth to explore how well the workshop process attended our overarching aims.

## Results

### The rich picture

**Fig. 1:**
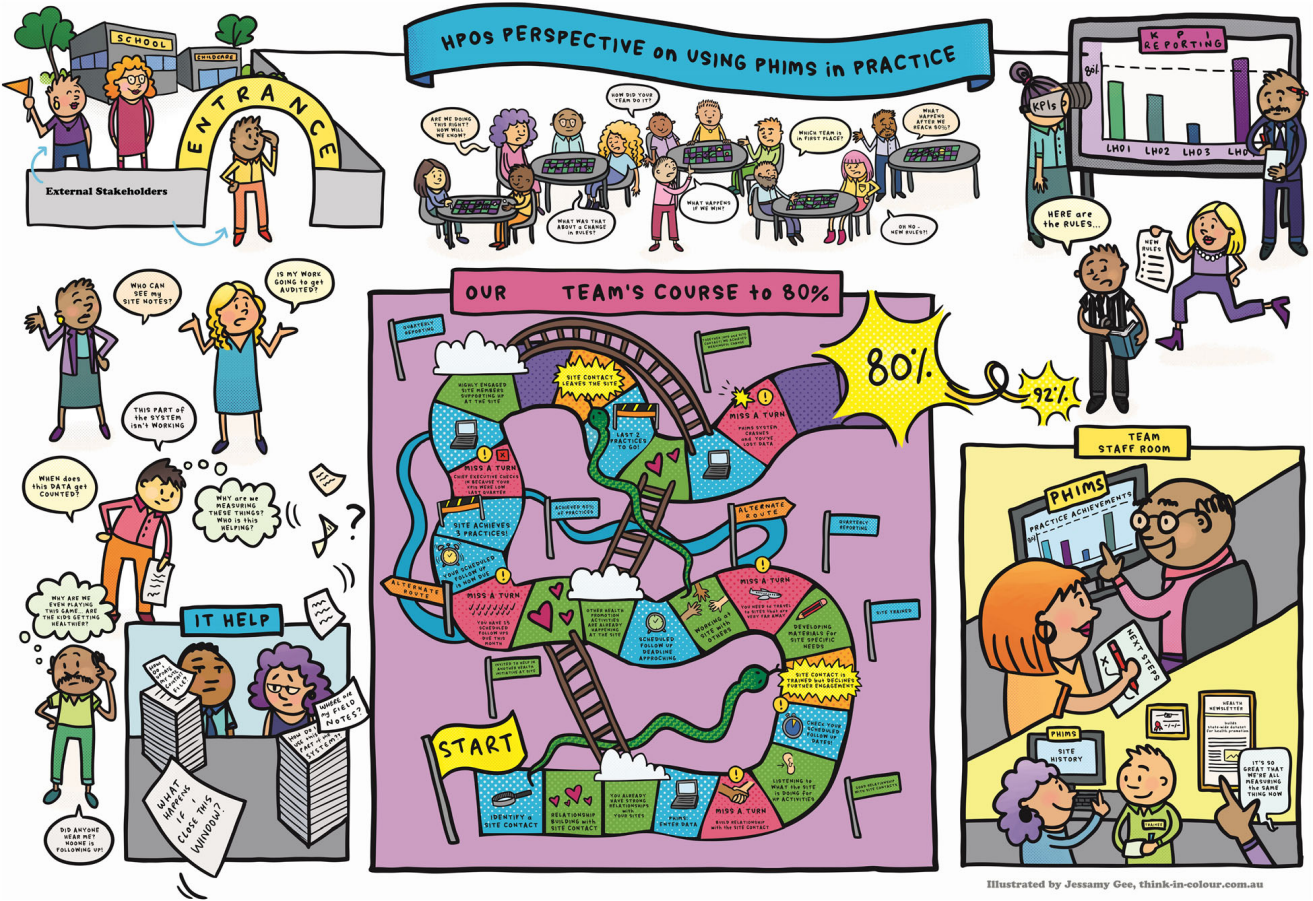
Rich picture of the Population Health Information Management System (PHIMS) of practice. *KPI* key performance indicators, *HPOs* Health Promotion Officers. Programme and stakeholder names have been removed to protect privacy

The rich picture is presented in Fig. 1. The metaphor depicted at the centre of the visual was a board game, drawing from a combination of the popular children’s games of ‘snakes and ladders’ and Candyland™. We identified these games progressively over the course of analysis. For example, while discussing data about practitioners’ experiences with PHIMS, we observed that this was like a game of chance where progress was often beyond the players’ (i.e. practitioners’) control and appeared to be ‘two-steps forward, one-step back’. This reminded us of our experiences playing ‘snakes and ladders’. Therefore, we chose these games for their ability to depict how practitioners advance work goals by accomplishing specific programme and PHIMS-related activities and because they provided a familiar reference point. The game metaphor reflects the key findings labelled ‘1. Parts of the PHIMS System’, as outlined in Table 3, where the sub-categories are represented as game components. Findings 2.2–2.4 in Table 3 illustrate dynamics between parts of the system. Incongruencies between PHIMS demands and health promotion work, how practitioners deal with these incongruences, and where PHIMS could help better support practice are intentionally implicit in the game. We aimed to provide a balance between a concrete representation of common events occurring in the system and these incongruences to invite reflection and consideration of how the system could be improved.

At the bottom right of the visual are depicted examples of how PHIMS is a helpful tool in supporting health promotion work. This reflects finding 2.1 about how PHIMS is used. Around the central game board are health promotion practitioners voicing questions and confusion about how best to use PHIMS. This content reflects categories 2.5 & 2.6 that deal with themes regarding practitioners’ concerns and confusion about how PHIMS data is used and difficulties experienced using the PHIMS system. At the top, health promotion teams are playing their own game boards, but questioning the overarching rules of the game. This reflects finding 2.7 – fears or concerns regarding the use and purpose of PHIMS. To provide context, we depict key stakeholders who are observing the game and state-level programme managers who monitor progress. Collectively, this material also reflects various roles of people who make up the PHIMS system.

The placement of these stories is purposeful, with each area of the visual representing key patterns that emerged from the data (as outlined in Table 3). The content that makes up the characters’ thoughts are not verbatim quotes but are synthesised from the data.

### Workshop activities facilitate sensemaking

The interactive workshop was the process by which participants engaged in interrogating and unpacking the rich picture (i.e. second-level sense making). Each activity introduced questions to provoke curiosity and new insights about how the PHIMS system operates and thereby to expand the transparency of the system itself. The process and design of the workshop was just as critical to the inquiry as the picture itself. Participants were divided into small groups comprising a mixed representation of teams and roles. Each group explored three related themes through a series of unique questions (Appendix 2 in Table 6). We report on the broader themes and how they prompted sensemaking below.

### Picture theme 1: why do you think the characters are asking these questions?

These questions directed participants to engage with specific parts of the rich picture. They prompted participants to consider multiple interpretations behind why the characters were asking questions about the aspects of the system and how it works. We observed that participants drew heavily on their personal experiences and knowledge to bring in new information about other perspectives that they did not see reflected in the picture, for example, naming individuals and conversations that would contradict some of the information represented in the picture. They related their own perspectives to those depicted and used these insights to examine what new information might help to resolve unanswered questions. Both groups questioned who is or should be responsible for answering the questions depicted. As seen below, this led to conversations about roles and responsibilities of agents in the systems, inquiries into how communication flows throughout the system and discussions about possible blockages and their causes.

Conversation excerpt from group 2:

Programme manager 1: *That’s what stuck out at me on that map there, is you know- they immediately go to ...*

Programme manager 2: *Each other* [said simultaneously]*.*

Programme manager 1: *Each other* [to ask questions] *instead of going straight to you know the ...*

IT designer 1: *Yeah.. the oracle* [laughs, likely referring to their team as the IT experts].

Programme manager 2: *... so either they’re scared to go to* [us] *or they don’t know – I don’t know, that’s the question.*

### Picture theme 2: what do you notice about the relationship between health promotion practice and PHIMS?

These questions directed participants to examine the dynamics between different components of the picture. Participants’ sense-making discussions identified unintended consequences of the design of the system. Both groups identified that the health promotion teams seem to be in competition with one another and explored whether this competition could be driven or reinforced by the PHIMS IT programme itself. For example, one group explored how follow-up reminders built into PHIMS seem to drive activities to the detriment of health promotion practice, although the original design intention was that this feature only support practice:

Conversation excerpt from group 1:

Programme manager 3: [pointing to the picture]*… it’s all about the system dictating schedule follow ups, rather than, you know, meeting with the school, trying to engage and then recording in the system. It’s about the system is driving us to do scheduled follow ups... I think best practice health promotion is that you’re working with the site. This scheduled follow-up is the mechanism for organising that … .*

IT designer 2: *That should be it. If you strip* [it down, the follow up reminder is] *just a mechanism. Yes. Just a mechanism, rather than a guideline.*

Programme manager 4: *But I think it’s evident they don’t understand that, proving what we’re saying. It seems to be a competition* [between the teams] – *they’re competing to do that follow-up at the expense of their generalised health promotion.*

The other group discussed how the target tracking and reporting functions of PHIMS – although originally designed to track progress towards impacting childhood obesity rates – is being used as a system to monitor staff performance.

Conversation excerpt from group 2:

Programme manager 1: *… but then we also know that* [PHIMS is] *being used for personal performance.*

IT designer 1: *Oh?* (interested/surprised).

Programme manager 1: *So, the health promotion directors, supposedly, are looking at how their staff member’s sites are achieving, so that could be a reflection on the staff member.*

It designer 1: *Wow.*

Programme manager 1: *Yeah, so that’s another …* [Name removed] *brought that up saying ‘well, there’s another aspect that you guys haven’t considered, that we monitor the staff performance using the system.’ And that’s really hard for schools* [to implement this programme]*, but it’s not necessarily the* [health promotion] *staff members. It’s that the school is not engaged...*

IT designer 1: *Wow, you know, that’s really interesting. I was reflecting on this about the unintended consequence of the* [performance monitoring] *framework, and this idea of useful competition versus unhealthy competition. I don’t think we’ve really explored that in enough detail to be able to put in mitigation.*

Programme manager 1: *Right, there was never … We never saw it as the purpose of the system, but we know that some teams are using it that way.*

The excerpts above also reveal that participants generated new insights about PHIMS from their shared sense-making process as much as from the rich picture itself. The picture provided an opportunity for them to explore different interpretations and share knowledge.

### Picture theme 3: what do you notice about the overall operation of the PHIMS system?

Questions within this theme aimed to encourage participants to reflect on the whole system, what the experience is like to work within it and what might be at stake should it not function the way it was intended. Participants reflected that the practitioners’ experience of PHIMS did not always align with their expectations and identified areas for improvement. They explored how the overall system of practice has changed over time; however, the PHIMS IT programme has not:

Conversation excerpt from group 2:

IT designer 1: *Well, it sounds to me that they’re trying to ‘game’ the system.*

Programme manager 2: *That might not be a bad thing. That might be that the system was developed- could have been developed in a different way that might be more efficient, but we didn’t know that when we developed it...*

IT designer 3: *It could be that the practice has evolved. I think one of the things that came up for me was that we haven’t had a chance to revisit the underlying assumptions that informs the original design of the system, and there’s been a lot of staff churn, and the practice is likely to have changed.*

Programme manager 2: *Exactly.*

### Were workshop aims and goals achieved?

The ethnographic field notes of the event describe a sense of enthusiasm and excitement among the participants, particularly regarding the ‘unveiling’ of the picture, and a strong sense of participant engagement and dialogue throughout the workshop. The evaluation confirmed that participants found the workshop insightful (mean = 5.14), a constructive way to explore issues of ‘transparency’ around PHIMS (mean = 5.86) and a good use of their time (mean = 5.57). Participants were highly engaged in dialogue about the image. Because they were from different operational teams, they exchanged information and perspectives with one another about how the system works and identified a range of potential solutions and lines of inquiry for further exploration. In the follow-up survey, several participants reflected that their lingering impression of the workshop was that there were diverse views of the PHIMS systems and that better communication is needed between state and local-level sites. Several remarked that the research process was valuable for raising new insights and directions for future work.

### Unanticipated results

Our intent was for the picture to be used as a tool for sense-making – and not as an objective representation of finding(s). In other words, we endeavoured to create an atmosphere in which results were viewed through an interpretivist lens (e.g. where meaning making comes from each persons’ interaction with the visual) rather than a positivist one (e.g. where meaning is external to any one persons’ interpretation). Through a series of reflective tasks, many of the workshop activities aimed to orient the participants to engage with the picture from a place of inquiry and curiosity. Yet, almost immediately following the unveiling, participants began asking about the extent to which different visual parts reflected specific people or quotes from the data. We had to regularly remind them the intention was one of metaphor and interpretation, not of objective representation.

Notably, participants seemed to struggle with or avoided articulating how they saw themselves as part of the system. We did not purposefully direct them to but had anticipated it might come out as part of the discussion. Yet, overall, they recognised that a lack of clarity in roles and responsibilities was an overarching problem in the system itself. They identified new insights and areas for future work directed at other actors in the system (e.g. how health promotion teams could improve work flows or how managers could better advocate for PHIMS) rather than reflecting on their own roles and actions, and how this influenced system dynamics:

IT designer 2: *The other thing I wondered is whether it’s useful to have some kind of ... visual on the ecosystem to provide a more holistic view, and some consistent messaging about a model and context, because if you start with that, then you’re automatically kind of forcing people to talk, to surface their assumptions about their role in the big picture. I think this is under, like everyone understands that there is a bigger ecosystem, but we’ve never really documented it in a way that could trigger a conversation to say, ‘This is* [your] *role and* [our] *role’.*

Participants compared and contrasted how the visual representation fit with their understanding of the ‘system’. Sometimes, this involved questioning the extent to which the data and our interpretations were accurate. This seemed to be particularly the case when the conclusions they reached were uncomfortable or less-than-desirable. For example, when one group discussed that the PHIMS programme is driving practice more than desired, participants challenged this conclusion, drawing on anecdotes that refute this interpretation while also challenging the research design. Therefore, the inquiry was not always a comfortable task – some interpretations confirmed participants’ understanding of the system but other interpretations were confronting. Upon unveiling the picture, one participant’s first response was “*this is really scary*”*.* This response was unexpected given our attention to creating a safe space, including the choice of cartoon-like images for the visual; however, perhaps it also reflects a high-level of engagement in which participants engaged with the visual to the extent that they recognised potential personal and professional implications, even if these remained unvoiced.

## Discussion

In this paper, we have provided a detailed account about how we developed a rich picture of ethnographic data and used it to engage research partners in inquiry and reflexivity as a means towards coproduction of a research project. As illustrated in our methods, the process of generating the rich picture was just as rigorous as conducting a qualitative analysis. Although our aim in this paper focused on the rich picture as a coproduction tool rather than a way of presenting research findings for an academic audience, in the future, journals might publish such rich pictures alongside qualitative analyses. We found the picture to be a highly effective tool to invite participants into the inquiry process, and to walk them through a series of reflective tasks. The combined picture and workshop process enabled participants to engage in systems thinking – considering systems theory principles, including exploring roles of different actors in the system, dynamics between system components that influence behaviours, and emergent but unintended consequences of the IT design. As a result, the participants gained deeper insights into the data and the PHIMS system overall and began considering new inquiries that they might explore to better enhance their work.

Using imagery via a rich picture presented some benefits in terms of translating and co-interpreting data. We found that using the picture offered an opportunity to express emotion, relationships and nuances that are present in the ethnographic data, but are not always easily communicated through speech and writing. Language can be limiting – words can carry various connotations or implicit values that are not shared by all – thus impeding communication [13, 32]. While visuals also contain implicit meanings, they more fully allow individuals to explore their own interpretations and state these in their own words. Therefore, we found the visual to be a useful way to depict problems in the PHIMS system without having to explicitly define the problem ourselves. Another benefit of the rich picture is that it enables the entire system to be seen at once, with the individual pieces in relation to one another. As opposed to traditional research outputs that are presented in a linear format (e.g. presentations or papers) requiring the observer to follow the author’s journey, a visual allows the viewer to be in control of their own process of interacting with material and drawing their own conclusions.

Participants valued the shared experience of working differently. Participants took their experience back to their workplace, and the picture was subsequently shared with the full coproduction team as part of a broader reporting process. It gave two departments within the state government who play very different roles the opportunity to move beyond collaboration focused on solution-generating to explore different understandings of a complex system. Ideally, a follow-up with the coproduction team would examine how insights from this process were applied and/or evolved over time.

Applying systems thinking will be critical to building the capacity of actors to work with complexity. However, despite explaining the rich picture as a metaphor to think with, some participants struggled with whether the content was ‘valid’. This likely reflects different epistemological stances to research that are introduced and reinforced through disciplinary training (i.e. many of our participants had a health background which tends to privilege positivist paradigms). Thinking in a new paradigm is uncomfortable and challenging, and our participants’ struggle reflects this. Further, we reflected that participants’ discussions did not explore their own role in the system – one of our underlying intentions of the workshop. Our sense is that these struggles were due to introducing a new way of working and participants’ comfort with the kinds of reflective questions posed rather than the design of the picture or workshop itself. Resolving such discomfort requires time to establish trust and comfort with new ways of working [33, 34]. We present the rich picture as one such way to facilitate this evolution. Further, because visual interpretations are subjective, the process described here allowed participants to engage with content and interpretations on their own terms and did not force any particular interpretation or conclusion. This can be an advantage in that it allows participants space to handle confronting information which, over time and through subsequent engagement and reflection, they may be able to gradually move towards deeper understanding. While this approach may not work for all projects or findings, it is a useful strategy for making the small shifts that comprise the groundwork of broader, system-level change.

Likewise, this experience of working and thinking differently may help to shift future expectations about the coproduction relationship – it illustrates that, in coproduction, both researchers and partners are required to work differently. It signals to partners that they are active members of the research process, not simply commissioners of research. It also requires researchers to generate opportunities and processes that invite partners more fully into the research process.

## Conclusion

Working in coproduction requires trailing, capturing and sharing our process of working differently, not only the results of having done so. By reporting our experience of using rich pictures we provide a tool that others may use in their own coproduction work. Because we are dealing with complex problems there will never be one answer, but we can seek to ask better questions that deepen our understanding of complexity. The process of using rich pictures enabled both parties – the researchers and our partners – to ask better questions both in the context of research and in practice.

## Data Availability

The data are not publicly available due to it containing information that could compromise research participant privacy and consent.

## Appendix 1

**Table 5:** Design considerations of the facilitated workshop on the rich picture

• Orientate participants: speak to the nature of the data (ethnographic) and where it has come from (the point of view of the ethnographer who was working alongside Health Promotion Officers)	
• There are significant power imbalances between what we surface and where it comes from (i.e. the people from whom the data was generated are not participants in this workshop), and between actors in the room ○ Should parts of the workshop be in silence? This is a way to attend to power differences ○ Acknowledge the absent actor in the system to make sure they are recognised and thought about during the day	
• Need to create a non-judgement-based approach to this data interrogation – need to work from a place of curiosity to allow for improvements, not judgements	
• Acknowledge this is not an exhaustive review of all issues – we hope to land on improvement ideas but will allow for flexibility in case participants need to spend more time in understanding versus problem solving	
• Potential concern that the participants may get into a group think, by defending their point of view against the picture	
• Logistics: ○ Half-day workshop with morning tea served ○ Large room with tables and chairs for 40 people, audio/visual equipment and white boards; tables can be moved or removed as needed ○ Stationary, including sticky notes, markers, tape and ability to display the rich picture are available	

## Appendix 2

**Table 6:** Workshop outline and guiding questions

Activity and guiding questions	Purpose
1. Introductions – 15 min	Familiarise everyone with who is in the room and their role
2. Preparatory work to grounding inquiry in curiosity Group dialogue: - What brings us into the room? - Bring this back to the question of inquiry that has driven this activity - Why are we breaking from a traditional approach?	We need them to be situated in curiosity, but this has to emerge from them; asking about how we came to be in the room can recall their interest in deeper inquiry, their own curiosity and why we are taking a different tack They will be able to speak to why they want to engage in this process
3. Systems inquiry intro – 10 min - What does it mean to inquire into a system? How do we do that? - Why visualisations are useful?	Introduce systems thinking concepts and provide a shared framework and language for workshop
4. Generation of the picture and nature of the data – 10 min - What is the nature of the data we are working with? - How did we work with it?	Explain how the picture was developed and acknowledge the limitations
5. Overview of approach and purpose of the day – 10 min	Set expectations
6. Drawing out mental models and assumptions – 20 min • How does PHIMS work, what does it do? - Participants draw a schematic diagram/concept map of the system • What do you know, what don’t you know? - Participants use a pre-prepared diagram to reflect on their knowledge	Unpack assumptions and biases participants bring with them about the PHIMS system and consider what new information might be useful
7. Discernment vs. judgement – 10 min - What is the difference between discernment and judgement? - Discern – identify a distinction between things - Judge – to apply value or merit to a thing	Expectation-setting that we aim for discernment in these exercises In systems, we want to discern patterns, relationships, dynamics; to pass judgement is to make assumptions
8. What don’t you know – put picture on the wall – 35 min Use different colour sticky notes to map different perspectives – done as a collective to better understand the system as a whole In silence, review the picture and get familiar 1) Review what is it you don’t know, state as a question – post on wall (colour 1) 2) Everyone reads the questions 3) Does the picture provide insights to any of the questions posted? What insight do you see on the picture that helps to answer a question posed? (colour 2) a) What additional insights can you offer from your own experience/perspective? (colours 3 and 4 – each team has a different colour)	This allows participants to explore the picture, but with a boundary (using what they’ve acknowledged they don’t know) We need to differentiate the perspectives of the participants from the perspectives depicted on the picture AND we don’t want to introduce questions beyond what the picture can speak to; scale is focussed on PHIMS as a whole, not about a specific part of the picture
9. Guided inquiry and interrogation of incongruences depicted in the picture – 60 min Three rounds of questions (20 min/round) 1) Form two teams 2) Each team to receive a question 3) For each, have individuals write their responses on sticky notes in silence, and read each other’s notes 4) After ~ 5–10 min, groups to discuss together 5) Answer prompt question: ‘Given what we’ve discussed, what might we …’ Prompts include what might we want to know, explore, do, ask … Three themes: 1) Why do you think the characters are asking these questions? 2) What do you notice about the relationship between health promotion practice and PHIMS? 3) What do you notice about the overall operation of the system?	Generative – gathering as much insights as possible See the issues JUST through the eyes of the health promotion officer Questions for Group #1 Why do you think the people near the IT information desk are asking these questions? (Theme 1) What do you notice about scheduled follow-up visits? (Theme 2) What do you think the health promotion officers’ experience of PHIMS is like? (Theme 3) Questions for Group #2 Why do you think the people seated at the game tables are asking these questions? (Theme 1) How does PHIMS support health promotion? (Theme 2) What happens if the rules of the system are not well understood? (Theme 3)
10. Reflect on new knowledge and new questions for inquiry – 10 min Reflect on what you didn’t know, can you develop new questions for future inquiry?	Captures new questions and insights that have emerged for inquiry that are more refined and informed

## Abbreviations

IT: Information Technology
PHIMS: Population Health Information Management System
SSM: Soft Systems Methodology
